# Study protocol: Addressing evidence and context to facilitate transfer and uptake of consultation recording use in oncology: A knowledge translation implementation study

**DOI:** 10.1186/1748-5908-6-20

**Published:** 2011-03-14

**Authors:** Thomas F Hack, J Dean Ruether, Lorna M Weir, Debjani Grenier, Lesley F Degner

**Affiliations:** 1 Faculty of Nursing, University of Manitoba, Winnipeg, Canada; 2 CancerCare Manitoba, Winnipeg, Canada; 3 Tom Baker Cancer Centre, Calgary, Canada; 4 Faculty of Medicine, University of Calgary, Calgary, Canada; 5 British Columbia Cancer Agency, Vancouver, Canada; 6 Faculty of Medicine, University of British Columbia, Vancouver, Canada; 7 Department of Internal Medicine, University of Manitoba, Winnipeg, Canada

## Abstract

**Background:**

The time period from diagnosis to the end of treatment is challenging for newly diagnosed cancer patients. Patients have a substantial need for information, decision aids, and psychosocial support. Recordings of initial oncology consultations improve information recall, reduce anxiety, enhance patient satisfaction with communication, and increase patients' perceptions that the essential aspects of their disease and treatment have been addressed during the consultation. Despite the research evidence supporting the provision of consultation recordings, uptake of this intervention into oncology practice has been slow. The primary aim of this project is to conduct an implementation study to explicate the contextual factors, including use of evidence, that facilitate and impede the transfer and uptake of consultation-recording use in a sample of patients newly diagnosed with breast or prostate cancer.

**Methods:**

Sixteen oncologists from cancer centres in three Canadian cities will participate in this three-phase study. The preimplementation phase will be used to identify and address those factors that are fundamental to facilitating the smooth adoption and delivery of the intervention during the implementation phase. During the implementation phase, breast and prostate cancer patients will receive a recording of their initial oncology consultation to take home. Patient interviews will be conducted in the days following the consultation to gather feedback on the benefits of the intervention. Patients will complete the Digital Recording Use Semi-Structured Interview (DRUSSI) and be invited to participate in focus groups in which their experiences with the consultation recording will be explored. Oncologists will receive a summary letter detailing the benefits voiced by their patients. The postimplementation phase includes a conceptual framework development meeting and a seven-point dissemination strategy.

**Discussion:**

Consultation recording has been used in oncology, family medicine, and other medicine specialties, and despite affirming evidence and probable applications to a large number of diseases and a variety of clinical contexts, clinical adoption of this intervention has been slow. The proposed study findings will advance our conceptual knowledge of the ways to enhance uptake of consultation recordings in oncology.

## Background

The time from diagnosis through to completion of therapy is challenging for newly diagnosed cancer patients. Patients have a substantial need for information, decision aids, and psychosocial support. One intervention that has proved promising in addressing these needs is providing a recording of the initial consultation [1-16]. Consultation recordings allow for memories to be refreshed, for the learning of information not recalled from the initial consultation, for a clearer understanding of one's cancer treatment [6-8], for greater confidence that critical aspects of the disease and treatment have been discussed [1-3], and for greater information recall in comparison to nontape controls [5,10,14,15]. Consultation recordings also provide patients with a means to initiate treatment discussions with family members [4,7-10] and help patients assume a significantly more active role in subsequent consultations [6] and in treatment decision making [16]. Patients prefer consultation recordings over standardised, prerecorded consultations [13] and general summary letters [12]. Consultation recordings are well received by the majority of cancer patients, and patient satisfaction with the intervention is high [1,2,4,5,10,12]. Reviews of the empirical evidence support the conclusion that recordings of oncology consultations improve information recall, reduce patient anxiety, enhance patient satisfaction with communication, and increase patients' perceptions that essential aspects of their disease and treatment have been addressed during the initial consultation [17-19]. The Cochrane Collaborative Group recently concluded that although more research is needed to improve our understanding of this intervention, the provision of recordings of key consultations may benefit most adults with cancer, and practitioners should therefore consider offering consultation recordings to patients [18].

Despite the empirical evidence supporting the provision of consultation recordings, the uptake of this intervention into oncology practice has not been widespread. A strong evidence base to support the broader use of this intervention is necessary but insufficient on its own for successful uptake. Knowledge translation (KT) theories are useful for understanding why the uptake of promising psychosocial interventions is slower than might be expected given the supportive evidence base and suggest that efforts towards wide-scale adoption should be delayed until the obstacles that impede uptake have been sufficiently identified and addressed.

### Aims

The overall goal of this implementation study is to examine the process of implementing consultation-recording practice into initial oncology consultations. Implementation studies are designed to examine in detail the processes involved in the transfer and uptake of an intervention and to identify and address the barriers to successful implementation. Guided by the Promoting Action on Research Implementation in Health Services (PARIHS) framework, diffusion of innovation theory, and social network theory, we will examine ways to transfer intervention knowledge and support intervention uptake, addressing the mechanisms that serve to retard or promote the transfer and uptake of this promising intervention.

Our team's earlier efforts confirm the utility of consultation recording use, but do not allow us to understand the factors that contribute to the derivation of patient benefit. If these factors can be identified, then this knowledge can be used to facilitate uptake of consultation-recording use in a manner that maximizes benefit. An additional goal, therefore, of this study is to follow patients more closely during the first few days following receipt of the consultation recording (*i.e.*, the time at which the recording tends to be listened to, to document the patient's perspective with respect to the specific benefits that are realised). For example, what information on the recording is most helpful to patients? Does the recording improve informed decision making? Is there a more intangible benefit to having a recording, such as being more positively disposed towards the oncologist or feeling more supported by loved ones after having family members listen to the recording?

The objectives of this project are three-fold:

1. To conduct an implementation study, guided by the PARIHS conceptual framework, diffusion of innovation theory, and social network theory, to explicate the contextual factors, including use of evidence, that facilitate and impede the transfer and uptake of consultation-recording use in a sample of patients newly diagnosed with breast or prostate cancer and the oncologists who treat them;

2. To systematically examine whether patients can articulate the benefits received from accessing their consultation recordings and to formally document these benefits for use in facilitating the most efficient--from a local health resource perspective--and effective uptake of the intervention;

3. To develop a conceptual framework that describes the interrelationship of evidentiary, contextual, and facilitative factors that is fundamental to the successful transfer and uptake of the consultation-recording intervention. Guided by the PARIHS framework, diffusion of innovation theory, and social network theory, the implementation study findings will be used to build this framework.

### Conceptual foundation

One of the more common findings from health-services research is a failure to routinely translate research findings into daily clinical practice [20]. Simple diffusion and passive dissemination of information are largely ineffective at changing practice [21]. Some practitioners have difficulty finding, assessing, interpreting, and applying the best evidence [22-24]. This problem has arisen, in part, from empirical information overload and the complexity of research findings [25]. As a result, clinicians look to someone else to perform these functions on their behalf. Even if ability permits these functions to be performed, there may be insufficient time to review the empirical research literature and other evaluative materials. While evidence is a fundamental consideration in the translation of new knowledge, transfer efforts are impeded by not considering KT more broadly. Patients, for example, are a stakeholder in the knowledge transfer process, yet they are frequently not regarded as such.

The PARIHS framework [26] proposes that KT can be explained as a function of the relationship between evidence (research, clinical experience, and patient preferences), context (culture, leadership, and measurement), and facilitation (characteristics, role, and style), with these three elements having a dynamic and simultaneous relationship [27-29]. The most successful implementation occurs when evidence is robust, when the context is receptive to change, and where the change process is appropriately facilitated [28].

**Figure 1:**
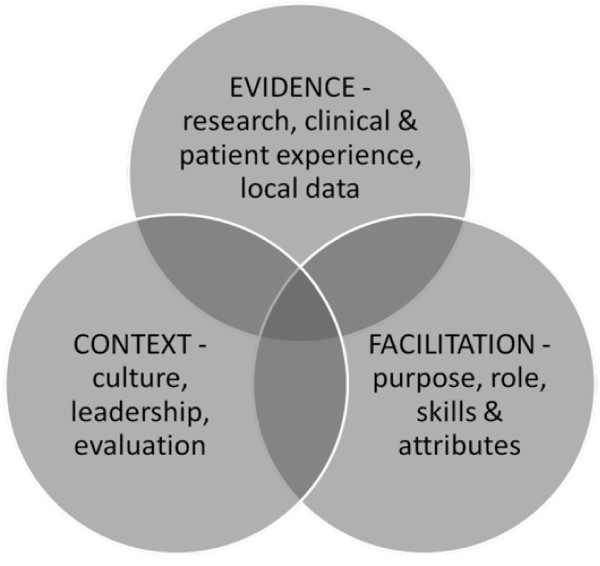
**PARIHS framework: interrelationship of evidence, context, and facilitation**.

The PARIHS framework, with revised subelements [30], is presented as Figure 1. The elements of the PARIHS framework have been shown in previous implementation studies to be robust and meaningful but should be systematically applied in each implementation study to ensure that the framework is appropriate, comprehensive, and accurate for the given contextual setting under study [31]. In accordance with a recent review that highlighted the importance of using the PARIHS framework in a prospective, rigorous manner [32], each of the components of this framework will be systematically examined and addressed during all phases of this implementation study.

According to the diffusion of innovation theory, diffusion is the process by which an innovation is communicated through certain channels over time among members of a social system [33]. The goal is to understand the social network of the social system and intervene appropriately to facilitate innovation knowledge, positive-attitude formation, and intent to use the intervention so that implementation will be successful. In our study, our goal is to educate oncology staff about the existing empirical base of support for consultation-recording use and to address the system elements and processes, identified in this theory, that are known to influence the rate of adoption: perceived relative advantage of consultation-recording use, compatibility of consultation-recording use with existing staff values and perceived patient needs, complexity of use of the consultation recordings, and the opportunity to try out the intervention (divisibility). Knowledge transfer is an important primary component of intervention adoption, and successful implementation includes reinforcement of implementation. This study will address the question of whether sharing with oncologists the benefits received by their patients from having a consultation recording has a reinforcing influence on consultation recording use by oncologists?

### Interplay of evidence, context, and facilitation

Motivation is a critical behaviour-change factor that underlies the use of consultation recordings by oncology professionals. Lack of exposure to the benefits of consultation recordings may result in clinicians who believe there is a lack of positive, consensus evidence for their use. Where unfounded negative attitudes towards this intervention exist, these attitudes may serve as heavy barriers for implementation. For this reason, efforts to educate oncologists about the benefits of consultation recordings may be a fundamental component of oncologist acceptance of the intervention and successful implementation. Where positive oncologist attitudes towards consultation-recording use exist, those who form an intention to do so may later forget or lose interest in doing so if they are not reminded or the intention is not otherwise reinforced.

Although many important barriers to KT exist at the level of the healthcare professional [26], there are multiple barriers to integrating research evidence into practice, many of which operate at levels beyond the control of the individual clinician. These include structural, organisational, and social barriers. Structural barriers are those environmental factors that impede KT. In oncology clinics, a frequently occurring structural barrier to adoption of psychosocial interventions is a lack of financial resources: Consultation recording equipment must be purchased and staff resources may be necessary to enable implementation. In the absence of monetary support, it may not be possible to disseminate this intervention. A potential organisational barrier is the absence of institutional or collegial peer pressures to use this intervention. The likelihood of uptake of consultation recordings may be enhanced through the support of the Chief Executive Officer of the cancer centre or of one or more oncologists who can serve as 'champions' in encouraging the use of consultation recordings.

Social barriers are often critical when groups of individuals are encouraged to adopt an intervention. The successful uptake of consultation-recording use may require a substantial proportion or critical mass of oncologists integrating the intervention into clinical practice. Social network theory predicts that an intervention is more likely to be adopted the greater the number of individuals who use it and if an integrated social structure can be established to support adoption [34]. By deliberately rewiring the interactions between oncologists, nurses, and patients through consultation-recording use, we aim to increase the density of the cancer patient-professional social network. Social network theory also suggests that those individuals with the most influence or power in using the intervention and promoting its use among others should be identified as change agents. Among oncologists, disease site leaders might be identified and approached, particularly if these oncologists can lead other oncologists within their disease specialty to adopt consultation-recording use. Key oncologists in support of consultation-recording use have been identified in each centre and invited to join the research team as coinvestigators. These oncologists (Grenier, Ruether, and Weir) have previously used consultation recordings in clinical practice and hold senior positions within their respective cancer disease sites. They are well suited, therefore, by their practice history and power status to serve as local champions for the use of consultation recordings.

An implicit assumption in much of the writing is that most KT activities should be directed toward the clinician. There are proportionately fewer studies that identify selected patient groups as the target for change. This is perhaps not surprising given that the goal of most knowledge-transfer activities is to change the practice style of treating physicians. However, there may be evidence that is sufficiently compelling to convince a significant portion of the general public to mobilise in an effort to change the course of clinical practice. The role of cancer patients and their advocacy organisations in promoting interventions that may enhance their psychological well-being should not be underestimated. Cancer advocacy networks may be powerful allies in promoting the dissemination of interventions that may be intimately tied to their emotional welfare. Interventions like consultation recordings that enhance cancer patients' feelings of control as they traverse the challenges of their disease trajectory are supported by patient advocacy groups. These groups have been known to exert powerful and positive influences on the media, government, and research funding bodies. To ignore patient advocacy groups is to create an additional barrier to successful KT. For the proposed implementation study, the role of cancer patients and their advocacy groups as change agents in our KT efforts will be identified and addressed. The use of patients as 'change agents' will be explored by systematically documenting the benefits they derive from the recorded consultation and passing this feedback on to the oncologists and nurses by way of a thank you letter from the patient.

## Methods

### Prestudy assessment of contextual readiness to the proposed implementation study

An integrated approach to KT should involve a systematic process of discovering all the potential barriers and facilitators to adoption of the promising intervention, and a detailed evaluation of the resources required to overcome each of these barriers and set the facilitators in action. The KT literature strongly advocates that those people from within an organisation who are targets for uptake of the intervention, and those who can influence uptake, should be involved in developing strategies for change [35]. Consistent with the functions of knowledge 'brokering', if the translation goal is to see more clinicians using a new intervention, then the probability of success will be enhanced if clinicians are included as coinvestigators of the research and if they are involved in an advisory capacity throughout the research process [36]. For the proposed implementation study, therefore, a prestudy assessment of contextual readiness was conducted to gather opinions on consultation recording use in the sites under consideration for inclusion in the implementation study.

The principal investigator traveled to each centre to interview oncologists and front-line staff about consultation-recording use, asking them to share opinions on the relative merits and perceived barriers and facilitators of this intervention. Given that an understanding and acceptance of the best empirical evidence in support of consultation-recording use is fundamental to successful uptake, the interviewer arrived at each interview with evidence in hand: a copy of the Cochrane Collaboration systematic review of consultation-recording use, copies of the publications of the consultation-recording studies conducted by the research team, and a copy of a local newspaper article speaking to the value of consultation recordings for newly diagnosed oncology patients. The interview findings showed that the oncologists and nurses were able to identify several barriers and contextual factors that explained the current use of consultation recording in their centre. The respondents frequently differed both in their assessment of the benefits to patients of receiving a consultation recording and in their identification of the contextual factors that are critical to enhancing uptake of consultation-recording use. All of the interviewees supported an implementation study and expressed their willingness to participate in the study, for which the methods are described below.

### Oncologist/nurse sample

The oncologists who have signed letters of agreement to participate include six radiation oncologists from the British Columbia Cancer Agency, Vancouver, Canada who specialise in breast cancer treatment; six medical oncologists from the Tom Baker Cancer Centre in Calgary, Canada who treat prostate cancer; two surgical oncologists from the Breast Health Centre in Winnipeg, Canada; and two medical oncologists who treat breast cancer at CancerCare Manitoba in Winnipeg, Canada. The oncologists for the implementation study were purposively chosen to satisfy six criteria. First, only a handful of sites are necessary for an implementation study because the goal is to extensively examine the *process *of implementation rather than generate a large volume of data to enhance statistical power. Second, the goal is to have the participation of an oncologist base that treats a majority of the patients with a given cancer type at each location. Third, the goal is to selectively sample a few locations to obtain sufficient breadth of contextual and facilitative factors in the organisation and delivery of cancer services. Breadth of factors is important in an implementation study whose aim is to be as collectively exhaustive as possible in the identification of barriers and facilitators of the intervention. For this reason, three Canadian cities (4 locations) are included. Fourth, to enhance breadth, we will capture the consultation-recording experiences of surgical, medical, and radiation oncologists, and no two participating locations will have the same clinical subspecialty of oncologist treating the same cancer disease type. Fifth, to enhance uptake, our sample includes a mix of oncologists who did or did not participate in our earlier recording studies. Sixth, we are including breast and prostate oncologists because these are the two largest sex-specific cancer types, the cancer types for which the current consultation-recording evidence base is strongest, and with which the research team has the most prior experience.

### Patient sample

In the proposed implementation study there are two sampling aims. The first is to accrue a sufficient number of patients to allow for as complete a set as possible of consultation-recording circumstances that might arise for any given oncologist/nurse. The second aim is to accrue a sufficient number of patients per oncologist so that the use of the consultation-recording protocol will become smooth and routine, thereby facilitating behaviour maintenance. Patient inclusion criteria include the following: presenting with a primary diagnosis of nonrecurrent breast or prostate cancer, 18 years of age or older, and able to read and communicate using the English language. Patient exclusion criteria include brain metastases or other cognitive impairment that precludes provision of free and informed consent to participate in the study.

### Ethics

Ethics approval was obtained from the Institutional Review Boards at each of the three participating cancer centres, with the last approval letter received on July 5, 2010. All participating patients and health professionals (oncologists and oncology nurses) will sign consent forms. Interview and focus group notes will be anonymous. Participant data and a copy of each consultation recording will be stored in a lockable file cabinet for seven years after the end of the data collection period, at which time written materials will be shredded and recorded materials degaussed or destroyed.

### Procedures and materials

This study will be conducted in three phases: 1. preimplementation; 2. implementation; and 3. postimplementation phase.

#### Phase 1: preimplementation

The oncologists who are serving as local site coordinators will hire a research nurse associate (RA) for their particular location. A team meeting will then be scheduled to make any necessary modifications to the implementation protocol. The most important product of this meeting will be a detailed sequence of implementation steps that will be followed prior to launching the consultation-recording intervention in phase 2. The RAs will be invited to this meeting so they can meet each other and receive training. Subsequent to this team meeting and at quarterly intervals, the principal investigator (PI) will hold a conference call with the RAs to address any concerns and provide encouragement.

Prior to launching phase 2, the PI will travel to each location to introduce the study at either grand rounds or disease site rounds. The PI will then meet with each participating oncologist and primary nurse for a short, recorded interview. At the interview, the results of the prestudy contextual readiness assessment will be shared, the implementation plan will be reviewed, feedback with respect to the plan will be elicited, and the oncologist/nurse will be asked to sign an informed consent form. The PI will also interview other clinic staff--nurses, oncology residents, ward clerks, information technology personnel, privacy officers--to share the details of the implementation plan and record feedback. If any of these staff will be recorded during any of the consultations, then the RA will ask that they also sign an informed consent form. After the interviews have been completed, the local site coordinators, with support from the PI, research team, and RAs, will address the issues of evidence, context, and facilitation generated through these interviews. Only after the implementation considerations have been addressed will phase 2 begin. To capitalise on the implementation knowledge gained at each location, the phase 1 PI visit and oncologist interviews will begin at the second location only after phase 2 has been completed at the first location.

#### Phase 2: implementation

Although the precise procedures will only be known by completing phase 1, the general procedures will be as follows: The RA will identify, with the assistance of a ward clerk or clinic nurse, those patients of the participating oncologists that meet the eligibility criteria. The RA will contact the patient prior to the consultation day to explain the study and obtain verbal informed consent. The RA will check to see whether or not the patient has access to a computer for the purpose of listening to the recorded consultation. An analog recording will be offered to patients without access to a computer. On the day of the consultation, the RA will greet the patient in the waiting area to acquire written consent to participate and complete the Patient Sociodemographic and Disease Information Form. The RA will inform the oncologist or primary nurse that a patient who is participating in the implementation study has arrived and will ensure that the recording materials are in place. The exact delivery mode for the recording of the consultations will be tailored to each location during the implementation study. With audiotape use becoming obsolete, however, and based on feedback during the preliminary phase, we plan to record each consultation with a hand-held digital recorder and then immediately download the consultation to either a USB key (*i.e.*, memory stick) that is handed to the patient or a website for the patient to access with a passcode. Given that valuable information is commonly imparted to the patient by the primary nurse during the initial consultation, the consultation with the oncologist and the consultation with the primary nurse will be recorded. The RA will meet the patient after the consultation to provide a copy of the consultation recording or explain how she or he may gain access to the recording via computer. The RA will contact the patient two days postconsultation by telephone to find out whether the recording was listened to and to administer the Digital Recording Use Semi-Structured Interview (DRUSSI) [1,2] if this is the case. The patient will be contacted again at seven days postconsultation to administer the DRUSSI once more. The DRUSSI has evolved over successive research studies conducted by the team. Although we coined the term DRUSSI for the current proposal, we first used a variant of the DRUSSI (referred to then as the Audiotape Questionnaire) in the late 1990s in a pilot study for a larger randomised controlled trial (RCT) we conducted with women with breast cancer. For our two RCTs conducted with breast and prostate cancer patients, this instrument was called the Audiotape Use and Satisfaction Questionnaire. New to the DRUSSI for the current proposal are questions #2 ('When did you listen to the recording?'), #5 ('When did you make your treatment decision?'), and #6 ('Did the recording assist in making your treatment decision?'). The estimated maximum time for completion of the DRUSSI is 20 minutes. It is expected that each of the interviews (two days and seven days postconsultation) will not exceed 30 minutes.

Nearing the close of the final patient interview, the RA will invite those patients who listened to the recorded consultation to share their consultation-recording experience with the oncologist/nurse as a way of providing facilitative feedback. For willing patients, the RA will prepare a letter on behalf of the patient based on responses to the DRUSSI. The RA will make arrangements for the patient to suggest changes to the letter. The RA will ask the patient to sign the letter before the RA brings it to the oncologist/nurse (or the patient may choose to give the letter to the oncologist/nurse). Just prior to ending the interview, the patient will be invited to attend a focus group of the patients who participated in the implementation study at that location. This focus group will be held in a meeting room at the site within two months following completion of the final patient interview at each location, that is, in phase 3 (below). The goal of the focus group is to provide feedback and learn more about the patients' consultation-recording experiences. A local patient advocate for the disease site (breast or prostate) will be invited to attend the focus group in an effort to promote the intervention. The RA and the PI will lead the focus groups; each focus group will be recorded and transcribed, and the transcript will be reviewed to identify salient points. The local site coordinator will contact the oncologist and primary nurse after five of their patients have participated, for the purpose of finding out if the study is progressing smoothly, to document oncologist/nurse feedback on their participation in the study, and to address any concerns. The aim is to identify and remedy as quickly as possible any unforeseen events or circumstances that might hamper the successful implementation of the intervention. The local site coordinator will maintain a log book to keep a written record of these events and circumstances and the actions taken to address them.

#### Phase 3: postimplementation

At approximately two months following the participation of the final patient at each location, the PI will travel to the location to attend the weekly disease group (breast, prostate/genitourinary) meeting or a similar meeting at which the staff categories (oncologists, nurses, clerks) involved in the implementation are likely to be represented. The purpose of this meeting will be to solicit feedback regarding the successes and challenges realised during the implementation phase. This meeting will also be recorded, transcribed, and analysed for identification of salient points. The patient focus group described above and the oncology staff meeting will be held on consecutive days. The patient focus group will be held on the first day so that the salient points from the focus group can be shared with staff at the disease type meeting the next day.

At one month following the last of the postimplementation review meetings with oncology staff, a meeting of the research team will be held to review the findings of the postimplementation staff meetings, patient focus groups, and data from the DRUSSI and to complete a conceptual framework that describes the interrelationship of evidentiary, contextual, and facilitative factors that are fundamental to the successful transfer and uptake of the consultation-recording intervention. The development of the conceptual framework will be guided by the PARIHS KT framework, diffusion of innovation theory, and social network theory and will be the most important task of this meeting.

This team meeting will also serve to review, revise, and delineate the execution of the strategy for the dissemination of the study findings. At present, the team has developed a seven-point dissemination strategy:

1. Grand rounds: Present the results of the study (DRUSSI findings, findings from the patient focus groups and oncology staff review meetings) at grand rounds or a similar forum at each implementation site.

2. Café Scientifique: Most universities have information events that bring academics, clinicians, patients, and the public together. We will present our findings at these events at the Universities of Calgary, British Columbia, and Manitoba.

3. Peer-reviewed journal publication: Submit manuscripts of our research findings to peer-reviewed journals.

4. Media: With the support of the Communications Officers at the British Columbia Cancer Agency, the Tom Baker Cancer Centre, and CancerCare Manitoba, local and national print, radio, and television news media will be contacted. The popular media (television and magazines) will also be contacted in the hope of reaching as many of the target audience of potential cancer patients as possible.

5. Book chapter: Drs. Hack and Degner recently published a book chapter titled "Audio-recording important consultations for patients and their families--putting evidence into practice" [37] to help establish consultation recordings as a valuable clinical practice. The value of consultation recordings and the use of theoretical frameworks to address implementation challenges are conveyed in this chapter.

6. Conference presentations: Present the research findings at local meetings and national and international conferences.

7. Symposium: Organise a one-day symposium, at which the findings will be discussed with a broad mix of purposefully selected stakeholders (academics, clinicians, patients, patient advocates) to establish a national direction for research in the area of consultation-recording use in oncology.

### Data management and analysis

A relational database will be created using SPSS for Windows (Version 15; SPSS Inc., Chicago, IL, USA) to store quantitative data. This database; copies of all consultation recordings; all patient sociodemographic, illness, and interview data; and oncologist/nurse interview data will be stored on a central server at the PI's institution, with access limited to specific users at the discretion of the PI; this server is backed up daily. All recordings will be sent to the PI via a key-protected internet website. Patient names will not be stored in data files (hard copy records linking patient names with computer identifiers will be stored in a separate location). Regular meetings of the PI, data manager, project staff, and biostatistical consultant, if necessary, will be held to review progress and discuss concerns.

The sources of data for analysis will include oncology staff personal interviews and focus group interviews (preimplementation, implementation, and postimplementation), semistructured patient interviews (DRUSSI), and the patient benefit letters to the oncologists/nurses. All of the transcribed focus group interviews (patient and staff) and the individual staff interviews will be analysed according to the following qualitative analytic procedures: The study PI, local PI, and the local RA at each location will perform a latent content analysis of the transcripts for that location, coding the transcripts for common themes. Having three coders will help to minimize coding bias. A conference call involving the three coders will take place at approximately one-third of the way through the implementation phase at each site at each location for the purpose of achieving coding consistency of the data gathered to that point, discussing emerging themes, and deciding whether method changes are warranted, including possible modification of the DRUSSI (the telephone administration of the DRUSSI will preclude recording, but the RA will be instructed to take detailed notes that will be analysed and summarised into salient points). A second conference call will occur two-thirds into the implementation phase for the same purpose. A final conference call will take place after the last interview has been coded. The basic unit of coding will be a 'meaning unit', which may be a few words to a few sentences in length. Using open coding [38], the goal will be to arrive at 'distinct meaning units' by applying a constant comparative analysis [39,40] to new open codes that are applied, so that categories of similar codes are eventually generated. The next level of coding will be 'theoretical coding', whereby meaningful connections between categories are formed. Recordings will be transcribed as soon as possible, followed by the analysis of the transcripts within the following one to three weeks to enable timely modifications to method.

The development of the conceptual framework of consultation-recording implementation will occur during the postimplementation phase according to established criteria: Prior to the postimplementation team meeting, the PI and each local PI and RA (seven individuals in total) will work separately to generate the framework. With the analysis of the transcripts of the patient and staff qualitative data in hand, each individual will first use the PARIHS framework as a starting template to which the themes and text exemplars will be fitted. Where an obvious fit of the data to the framework does not exist, the framework will be modified or expanded. Second, each individual will fit the qualitative findings of the oncology staff data to the diffusion of innovation theory and produce a summary of how this theory can be used to explain the implementation of consultation-recording practice. Third, each individual will attempt to build a comprehensive explanation of consultation-recording use that incorporates the PARIHS framework, diffusion of innovation theory, and social network theory. At the team meeting, the draft frameworks generated by these seven individuals will be reviewed by the entire research team. The final product will be a comprehensive conceptual framework of consultation-recording transfer and uptake that has a solid theoretical and empirical foundation.

## Discussion

Implementation research has been criticised for failing to use implementation theories and frameworks in the development of protocols. A strength of this proposal is the prospective use of implementation theories, particularly the PARIHS framework, to identify, prioritise, and strategically address the contextual factors most relevant to successful implementation of consultation-recording use [32]. Particular attention has been paid to understanding the distinct contextual challenges in each of the cancer centres from which data will be gathered and tailoring the implementation strategy appropriately.

The proposed research will generate a conceptual framework for successful implementation of consultation-recording use in oncology, including a discussion of priority recommendations for implementation. A potential weakness of the proposed study is that the recommendations for successful implementation may be so heavily context specific as to limit the generalisability of the study findings to other oncology contexts. We will therefore endeavor to ascertain the key implementation factors that are generalisable to most oncology contexts and identify those specific factors that, while lacking generalisability across contexts, may be of critical relevance to particular contexts.

The impact of the proposed research lies beyond cancer. Consultation recordings have been used in family medicine and other medical subspecialties and have probable applications in many diseases and a variety of situations. The proposed study findings will advance our conceptual knowledge of intervention transfer and uptake and add to the field of KT.

## Competing interests

The authors declare that they have no competing interests.

## Authors' contributions

TFH is principal investigator on the project and prepared the initial draft of this manuscript. TFH, JDR, LMW, and LFD conceived of the study, formulated the study protocol, and were joined by DG in drafting the manuscript. JDR, LMW, and DG were instrumental in securing participation of oncologists. LFD shared her understanding of knowledge management theory. All authors read and approved the final manuscript.
